# Outstanding Enrofloxacin Removal Using an Unmodified Low-Cost Sorbent Prepared from the Leaves of *Pyracantha koidzumii*

**DOI:** 10.3390/antibiotics11111563

**Published:** 2022-11-06

**Authors:** Rubén Martínez-Escutia, Abraham Méndez-Albores, Alma Vázquez-Durán

**Affiliations:** Unidad de Investigación Multidisciplinaria L14-A1 (Ciencia y Tecnología de Materiales), Facultad de Estudios Superiores Cuautitlán, Universidad Nacional Autónoma de México, Cuautitlán Izcalli 54714, Mexico

**Keywords:** adsorption, agro-waste-based sorbent, enrofloxacin, aqueous media

## Abstract

Increasing discharges of synthetic antimicrobial agents from industrial and municipal sewage, as well as from agricultural runoffs into water bodies, is still a global challenge. Here, an unmodified low-cost sorbent was prepared in an ecofriendly manner from *Pyracantha koidzumii* leaves for the removal of enrofloxacin (ENR). Sorbent characterization was accomplished using Fourier transform infrared spectroscopy (FTIR), scanning electron microscopy (SEM), energy-dispersive X-ray spectroscopy (EDS), BET surface area, zeta potential, and point of zero charge. Biosorption assays were carried out via batch mode concerning the impact of adsorbent dosage, contact time, solution pH, solution ionic strength, adsorbate concentration, and temperature. In general, ENR adsorption was significantly correlated with pH and ionic strength. At a neutral pH, the sorbent had a theoretical maximal ENR uptake of 138.89 mg/g. However, the adsorption capacity was significantly affected by the presence of high concentrations of divalent cations (Ca^2+^ and Mg^2+^). The findings from the kinetics and isotherms showed that the pseudo-second-order kinetic and Langmuir isotherm models best fit the experimental data. Electrostatic interactions, hydrogen bonding, and π-π stacking were the most important mechanisms of adsorption of ENR onto the *P. koidzumii* sorbent. Overall, this study suggests the promising application of this agricultural residue for the efficient removal of ENR from water.

## 1. Introduction

Fluoroquinolones are potent broad-spectrum synthetic antimicrobial agents widely used in the treatment of bacterial infections. Enrofloxacin (ENR) or 1-cyclopropyl-7-(4-ethylpiperazin-1-yl)-6-fluoro-4-oxo-1,4-dihydroquinoline-3-carboxylic acid is a second-generation member of the quinolone family and was the first fluoroquinolone specially developed for veterinary purposes. This antibiotic is also extensively used in agriculture worldwide [1]. It is well known that, after fluoroquinolone administration, approximately 75% of the dose is excreted unchanged; thus, the nonmetabolized fraction can easily reach the environment, in particular, the aquatic ecosystem. ENR has been frequently detected at ng/L to µg/L levels in rivers [2], untreated drinking water [3], tap water [4], and surface and groundwater [5]. Although ENR seems to be easily degraded, this antimicrobial agent can persist in the aquatic ecosystem over a relatively long period of time, leading to the proliferation of antibiotic-resistant bacteria.

Due to the continuous discharge of antibiotics derived from anthropogenic (urban, industrial, and agricultural) activities, environmentally relevant concentrations of ENR in water bodies are often found; consequently, its degradation or removal by means of cost-effective and environmentally friendly methodologies is necessary. Several technologies, including ozonation [6]; reverse osmosis and nanofiltration [7]; electrochemical advanced oxidation processes, such as anodic oxidation with electrogenerated H_2_O_2_, Fenton, electro-Fenton, and photoelectro-Fenton [8,9]; photocatalytic degradation [10]; and adsorption [11,12,13,14] have been successfully used for the degradation/removal of ENR from water and wastewater. Of these methods, adsorption is widely used in industry to remove organic and inorganic pollutants. Adsorption has the advantage of removing contaminants totally or up to reasonable levels, and its efficiency depends mainly on sorbent structure (specific surface area, pore volume, pore size distribution, among others) and its chemical composition. In general, carbon-based materials are the most popular sorbents used to remove antibiotics [15]; however, their potential use at large scale is sometimes limited due to their high cost and regeneration difficulties. In order to overcome this situation, industrial or agricultural byproducts have also been proposed to produce low-cost adsorbents. As reported in the recent literature, several materials based on agricultural wastes have been effectively used for the adsorption of certain fluoroquinolones in aqueous media, such as ofloxacin [16,17,18,19], levofloxacin [20], ciprofloxacin [21,22,23], and norfloxacin [24]. However, the use of agricultural wastes for the removal of ENR, are still meager [25,26,27,28].

Agricultural solid wastes are all organics generated as byproducts from agriculture and gardening activities. Worldwide, the yearly estimated amount of agricultural waste produced is about 140,000 million metric tons [29]. Certain plant species are periodically pruned, and this activity generates substantial amounts of residues; such is the case of the invasive shrubs of the *Rosaceae* family. *Pyracantha koidzumii* (Hayata) Rehder is an evergreen shrub that finds it easy to adapt to a wider range of geographic environments; this, and several closely related species, are already present in numerous countries and produce considerable amounts of leaves. Thus, finding some uses for the pruning residues would convert a disposal problem into a value-added opportunity. Therefore, in this research, an unmodified sorbent prepared from *P. koidzumii* leaves was prepared for the removal of ENR in an aqueous solution. The influence of different factors, such as adsorbent dosage, contact time, solution pH, solution ionic strength, adsorbate concentration, and temperature, were evaluated to propose a novel and naturally abundant sorbent for ENR removal from water.

## 2. Results and Discussion

### 2.1. Characterization

**Figure 1:**
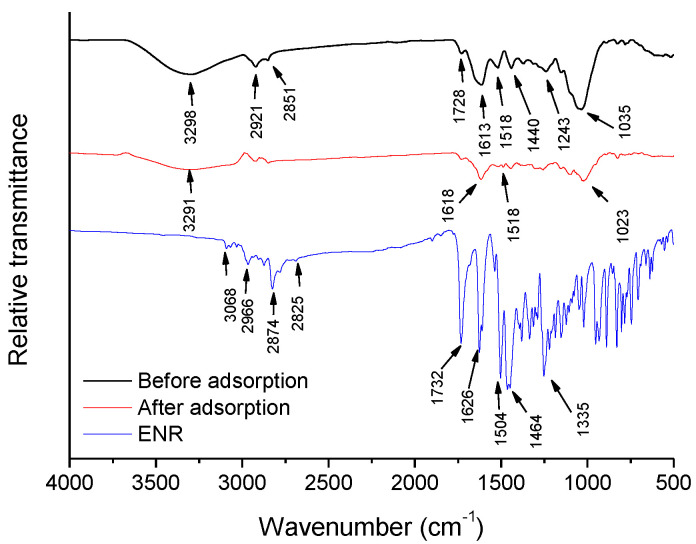
Fourier transform infrared spectra of the *P. koidzumii* sorbent (before and after adsorption) and the fluoroquinolone enrofloxacin.

Figure 1 shows the Fourier transform infrared spectroscopy-attenuated total reflection (FTIR-ATR) spectra of the unmodified sorbent (before and after ENR adsorption) and the fluoroquinolone ENR. In general, the sorbent showed a broad frequency band at 3298 cm^−1^, which is associated with free and intermolecular bonded hydroxyl groups, two medium frequency vibration bands of alkyl chains at 2921 cm^−1^ and 2851 cm^−1^, a weak vibration band of the C=O group of carboxylic acids or its esters at 1728 cm^−1^, and a strong vibration of the C=C stretching of aromatics at 1613 cm^−1^. The N–H bending of secondary aromatic amines and the CH_3_/CH_2_ antisymmetric bending of lipids and proteins appeared at 1518 cm^−1^ and 1440 cm^−1^, respectively. The medium band at 1243 cm^−1^ is characteristic of the phosphate antisymmetric stretch of DNA, RNA, phospholipids, and phosphorylated proteins, and the very broad frequency band located at 1035 cm^−1^ is related to the C–O or C–O–C stretches in polysaccharides [30]. Moreover, the proximate composition of the *P. koidzumii* sorbent is presented in Table 1. The results showed that carbohydrate (starch, sugar, cellulose, hemicellulose, and lignin) was the dominant macromolecular component, and protein was the second richest group. Thus, the sorbent contains plenty of –OH, C–O, and –NH groups, which was well corroborated by the FTIR measurements. For instance, the –OH band at 3298 cm^−1^, the C=O stretching vibration at 1728 cm^−1^, and the very broad frequency band at 1035 cm^−1^, which is ascribable to the C−O stretching vibration, confirm the presence of cellulose, hemicellulose, and lignin [31].

After ENR adsorption, significant modifications in the FTIR spectrum of the sorbent inferred the participation of certain functional groups in the process. In general, significant decreases in the relative intensity of the primary active vibrations and shifting to lower/higher wavenumbers were observed. As a result, the hydroxyl, carboxyl, aromatics, amine, and ether groups played a dominant role in ENR sorption (Figure 1). The FTIR spectra of the ENR showed weak O–H stretching vibrations, with super imposed C–H stretches at 3068, 2966, 2874, and 2825 cm^−1^, representative of the carboxyl and aromatic groups [32]. The strong vibrations at 1732 cm^−1^ and at 1626 cm^−1^ are commonly associated with the C=O stretching of the carboxyl and pyridine groups, respectively. The doublet at 1504 cm^−1^ and 1464 cm^−1^ confirms the presence of the aromatic C=C stretching vibration [33], and the band at 1335 cm^−1^ can be assigned to the C–N stretching mode of tertiary amines (Figure 1).

**Figure 2:**
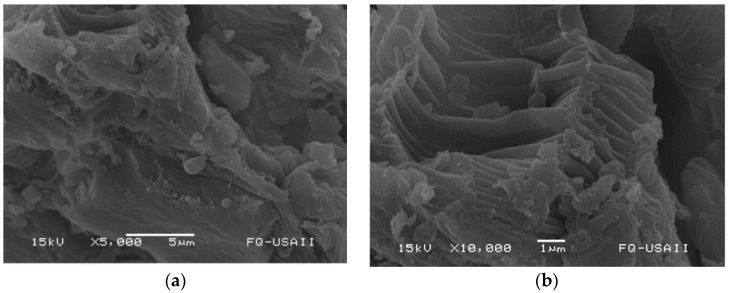
SEM-micrograph of the *P. koidzumii* sorbent. The upper part of image (**a**) is shown (**b**) at higher magnification.

The surface morphology and microstructure of the sorbent were evaluated using a series of acquired two-dimensional scanning electron microscopy (SEM) images (Figure 2). In general, the sorbent showed a corrugated structure like a washboard. The sorbent also possesses a rough and uneven surface. The gaps between these disordered uneven structures could allow better adsorption since these structures can provide suitable binding sites for ENR adsorption. Moreover, the elemental composition of the sorbent was determined by the energy-dispersive X-ray spectroscopy (EDS) technique, and the results are shown in Figure S1. The elemental mapping results confirm the existence of two main elements (C and O), accounting for up to 96.8% of the total weight of the sorbent. Other minor elements such as calcium (2.4%), magnesium (0.2%), sulfur (0.2%), potassium (0.1%), phosphorus (0.1%), aluminum (0.1%), and silicon (0.1%) were found to be evenly distributed on the surface of the material. The specific surface area of the sorbent was determined by the Brunauer–Emmett–Teller (BET) method. The BET surface area was found to be 0.9704 m^2^/g with a total pore volume of 0.00235 cm^3^/g. These results are consistent with SEM images that revealed that the surface of the sorbent is smooth with relatively low porosity. Similar results have been found in a sorbent prepared from teak (*Tectona grandis*) leaves [34].

**Figure 3:**
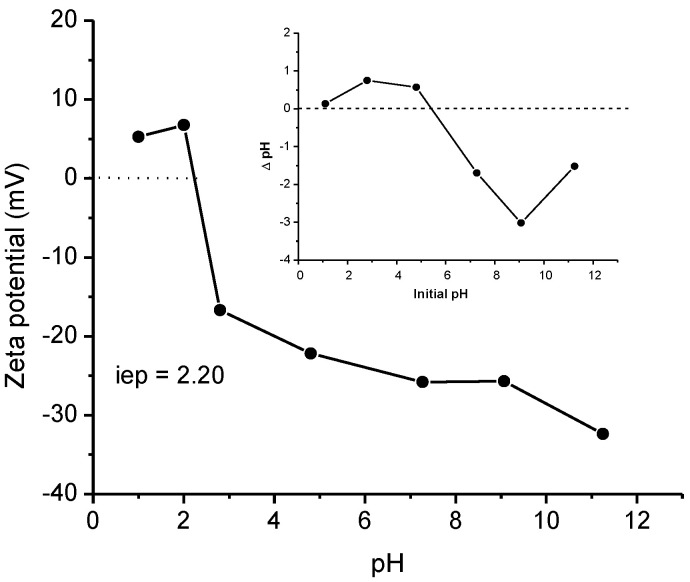
Zeta potential and point of zero charge of the *P. koidzumii* sorbent.

Figure 3 shows the zeta potential and pH_zpc_ of the sorbent. In general, in a pH range of 2.5–11.0, the zeta potential becomes more negative with increasing pH, reaching a maximum at pH 11 (−32.4 mV). The isoelectric point (iep) occurred in the vicinity of pH 2.2, indicating that the electrical potential at the slipping plane is zero. Furthermore, in the pH_pzc_ graphic—the plot inside the figure—the curve intersected the *x*-axis at pH 5.37, suggesting that the sorbent surface charge takes a zero value at this pH, which is in close agreement with our previous research [35].

### 2.2. Adsorption Studies

#### 2.2.1. Optimal Adsorbent Dosage

**Figure 4:**
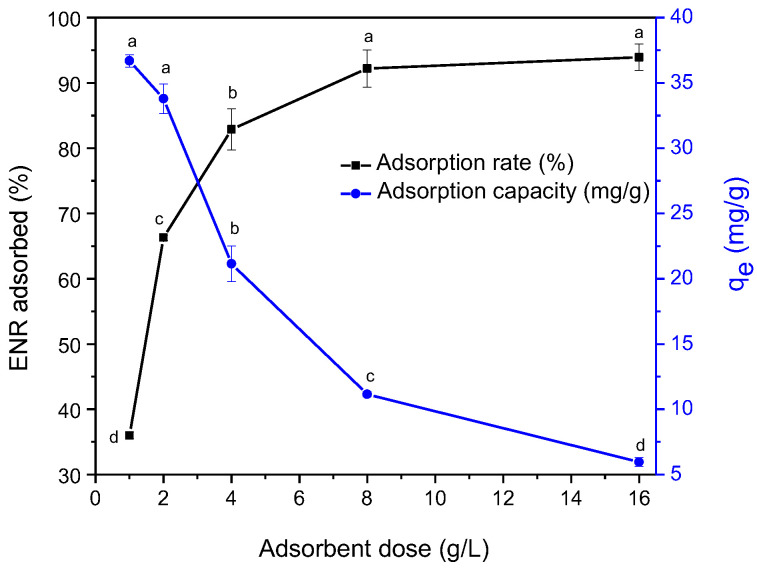
The effect of adsorbent dosage on the removal of enrofloxacin (contact time 1440 min; 100 mg ENR/L; pH 7; and temperature 298 K). For each parameter, those means that do not share a common superscript differ significantly (Tukey *p* < 0.05).

Figure 4 shows that significant increments in adsorbent dosage were positively correlated with the removal rate of ENR (because of the highly available surface area and the availability of the sites responsible for adsorbate uptake) but negatively correlated with the adsorption capacity ($q_{e}$). In general, the removal effectiveness increased gradually from 35 to 94% by increasing the adsorbent dosage from 1 to 16 g/L. The adsorption of ENR was enhanced with augmentation using sorbent doses up until 8 g/L (92%), but no significant change (*p* > 0.05) in ENR removal was observed at 16 g/L (94%) due to spatial inhibition of the adsorptive sites in the presence of excess dosage of the adsorbent [36]. With these considerations, the dosage of the adsorbent was set at 8 g/L ($q_{e}$ = 11.15 mg/g) for the subsequent experiments.

#### 2.2.2. Effect of Contact Time

**Figure 5:**
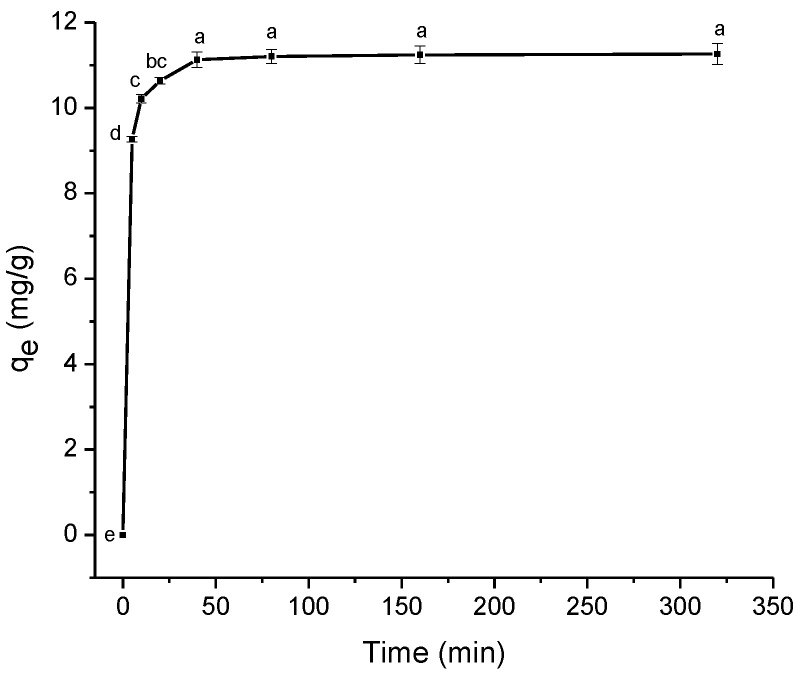
The effect of contact time on the removal of enrofloxacin (adsorbent dose 8 g/L; 100 mg ENR/L; pH 7; and temperature 298 K). Those means that do not share a common superscript differ significantly (Tukey *p* < 0.05).

The amount of ENR adsorbed was investigated as a function of contact time (0–320 min) under 8 g/L of adsorbent and a solution pH of 7 at 298 K (all conditions are listed in Section 3.4.1. The results are depicted in Figure 5. In general, at the beginning of the process, significant increments in the adsorption capacity were recorded because of the availability of more adsorptive sites; however, after 40 min, and up to 320 min, the adsorption capacity did not undergo significant changes (11.12 to 11.25 mg/g), indicating the equilibrium stage. Therefore, 40 min ($q_{e}$ = 11.12 mg/g) was selected as the optimum contact time for the subsequent experiments.

#### 2.2.3. Kinetic Studies

In the kinetic part of this research, three models (pseudo-first-order, pseudo-second-order, and intraparticle diffusion) were used to simulate the experimental data, and the governing equations are presented in their final form as follows:

(1) $$\ln\;\left(q_{e}-q_{t}\right)=\ln q_{e}-k_{1}t$$

(2) $$\frac{1}{q_{t}}=\frac{1}{k_{2}q_{e}^{2}}+\frac{1}{q_{e}}$$

(3) $$q_{t}=k_{p}t^{1/2}+C$$

In the models, $q_{e}$ and $q_{t}$ are the capacities of the sorbent to ENR uptake (mg/g) at the equilibrium and at time t (min), respectively. $k_{1}$ and $k_{2}$ are the pseudo-first-order and pseudo-second-order apparent adsorption rate constants (1/min and g/mg·min), respectively. $k_{p}$ (mg/g·min^1/2^) is the intraparticle diffusion rate constant, and C (mg/g) is a constant for any experiment.

**Figure 6:**
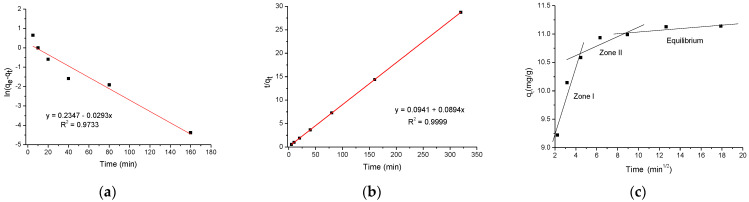
Kinetic models of enrofloxacin adsorption onto *P. koidzumii* sorbent (adsorbent dose 8 g/L; 100 mg ENR/L; pH 7; and temperature 298 K). (**a**) Pseudo-first-order, (**b**) pseudo-second-order, and (**c**) intraparticle diffusion models.

As can be observed, the pseudo-second-order kinetic model provided the best fit to the experimental data (Figure 6b). In general, the $q_{e}$ value (calculated) is in good agreement with the value obtained experimentally. Moreover, the coefficient of determination (R^2^) of the pseudo-second-order kinetic model was greater than that of the pseudo-first-order model (0.9999 vs. 0.9733). All kinetic parameters are listed in Table 2. Due to the close agreement between the experimental and calculated data, the pseudo-second-order kinetic model was selected to explain the ENR adsorption onto the sorbent. Thus, various mechanisms, such as surface adsorption and diffusion into the pores, participated mutually during the ENR adsorption process. Furthermore, to understand the mass transfer mechanism, the intraparticle diffusion model was applied. In general, the sorption data was fitted with three different linear zones/slopes (Figure 6c), and the R^2^ exhibited acceptable values (Table 2), indicating that the diffusion mechanisms were also influenced by the adsorption process. The first zone describes the mass transfer of ENR from the solution to the surface layer (film diffusion). The second zone refers to intraparticle diffusion of ENR on the internal pore surface. Furthermore, the slope for the first zone was sharper than the second one, indicating fast ENR transfer from the bulk solution to the sorbent surface at the initial time. After these two zones, a horizontal line was observed, indicating that equilibrium was reached.

#### 2.2.4. Effect of Solution pH

**Figure 7:**
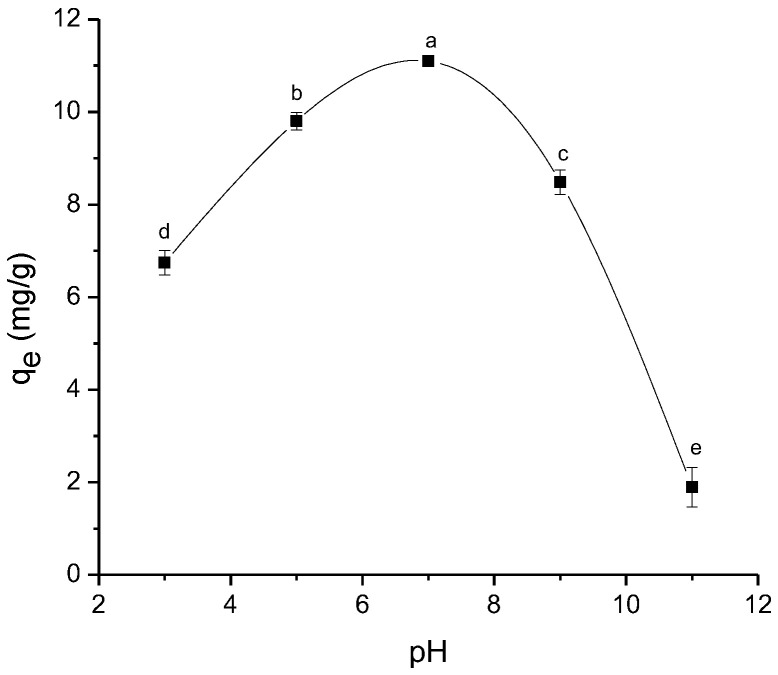
The effect of solution pH on the sorption of enrofloxacin onto *P. koidzumii* sorbent (adsorbent dose 8 g/L; 100 mg ENR/L; contact time 40 min; and temperature 298 K). Those means that do not share a common superscript differ significantly (Tukey *p* < 0.05).

Solution pH plays a key role in adsorption due to significant changes in the surface charge of the adsorbent and the protonation of the functional groups present in the adsorbate [37]. Figure 7 shows the effect of solution pH (from 3 to 11) on the adsorption capacity of ENR. Experimental conditions are fully described in the Materials and Methods Section 3.4.1. As observed, the adsorption capacity was mainly affected by the extreme pH values tested, but this effect was more notorious under alkaline conditions. In general, the adsorption capacity of ENR was reduced by up to 85% by increasing the solution pH from 7 to 11 (Figure 7). The reported pK_a1_ and pK_a2_ values for ENR were 5.95 and 8.70 [38]. These values corresponded to the carboxylic and the piperazinyl groups, respectively. It is well known that ENR can exist as cationic, zwitterionic, or anionic, depending on the pH value. At a pH ≥ 8.70, both the carboxyl and piperazinyl groups are in their ionized form (negatively charged), and the sorbent also had a high negatively-charged surface (Figure 3); as a result, low efficiencies against ENR removal were recorded because of electrostatic repulsive forces. Figure 3 also shows that the adsorbent has an isoelectric point of 2.20, meaning that the electric potential in the interfacial double layer is negative throughout the entire pH range (from 2.5 to 11). These findings clearly indicate that complex specific adsorption at the interface occurred with the cationic and zwitterionic forms of ENR [39]. In this research, as the maximum adsorption capacity was attained at a neutral pH (the natural pH of the solution), pH 7 was used for the adsorption of ENR in the next experiments.

#### 2.2.5. Effect of Solution Ionic Strength

**Figure 8:**
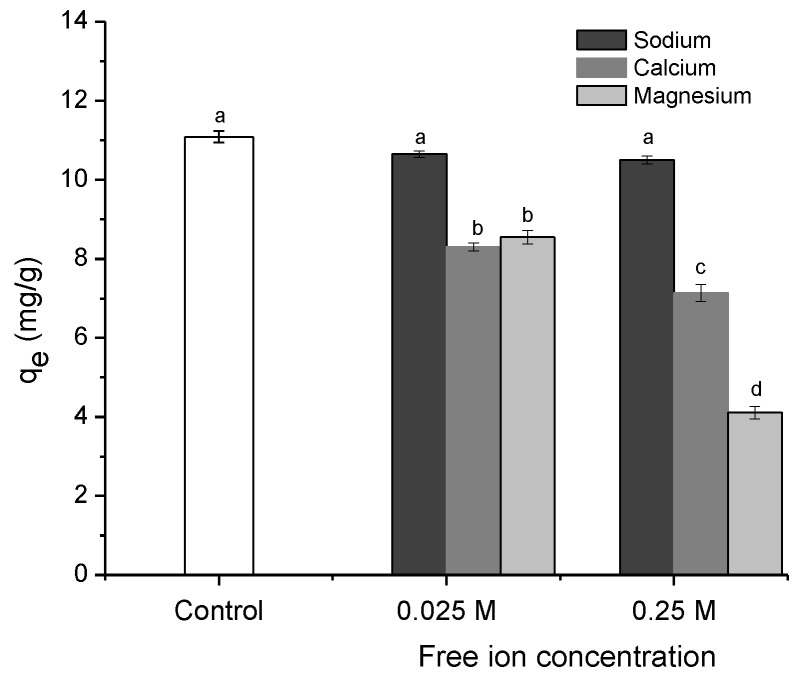
The effect of solution ion strength on the sorption of enrofloxacin onto the *P. koidzumii* sorbent (adsorbent dose 8 g/L; 100 mg ENR/L; contact time 40 min; pH 7; and temperature 298 K). Those means that do not share a common superscript differ significantly (Tukey *p* < 0.05).

The effects of three common salts (NaCl, CaCl_2_, and MgCl_2_) and their respective dissociated ions on the ENR adsorption capacity were investigated. The salts were evaluated at free ion concentrations of 0.025 and 0.25 mol/L. As displayed in Figure 8, Na^+^ (at both free ion concentrations tested) did not significantly affect (*p* > 0.05) the biosorption of ENR to any significant extent. However, at a free ion concentration of 0.025 mol/L, both the Ca^+2^ and Mg^+2^ ions reduced the adsorption capacity by 24%. Furthermore, as the divalent ion concentration increased to 0.25 mol/L, the adsorption capacity was significantly affected (up to 63%). The detrimental effect of the Mg^2+^ ions on the adsorption capacity of ENR was more pronounced than that of the Ca^2+^ ions suggesting the following: (a) similar binding sites, meaning competition between the Mg^2+^ ions and ENR for the adsorption sites, (b) a higher adsorption strength for the Mg^2+^ ions compared to ENR, (c) a charge shielding effect, or (d) the chelation between the divalent ion and the 4-oxo and the adjacent carboxyl group of the fluoroquinolone. Overall, the increase in the solution ionic strength only affected the adsorption of the ENR removed by adsorbate-adsorbent electrostatic interactions [40].

#### 2.2.6. Effect of Adsorbate Concentration

**Figure 9:**
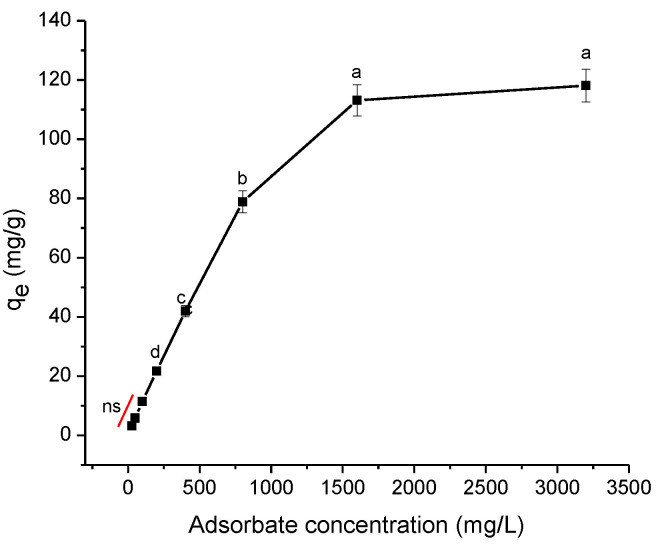
The effect of adsorbate concentration on the sorption of enrofloxacin onto *P. koidzumii* sorbent (adsorbent dose 8 g/L; contact time 40 min; pH 7; and temperature 298 K). Those means that do not share a common superscript differ significantly (Tukey *p* < 0.05). ns = not significant.

The influence of the initial adsorbate concentration on the adsorption capacity was also investigated, and the results are depicted in Figure 9. The experimental conditions are fully described in Section 3.4.1. As can be observed, the sorption capacity of ENR was rapidly raised by increasing the adsorbate concentration in the solution up to 1600 mg ENR/L. The amount of ENR adsorbed significantly improved from 3.17 to 113.16 mg/g when increasing the adsorbate concentration from 25 to 1600 mg/L, respectively. However, at the highest adsorbate concentration tested (3200 mg ENR/L), the sorption capacity did not differ significantly (*p* > 0.05). In general, the increase in the adsorbate concentration produced a stronger driving force to give more possibilities to the ENR molecules to interact with the adsorbent particles.

#### 2.2.7. Effect of Photosynthetic Pigments

A set of adsorption experiments were performed using a chlorophyll-free sorbent. Chlorophylls *a* and *b* were removed from the sorbent using 96% ethanol, and their contents were spectrophotometrically assessed, as described by Wintermans and De Mots [41]. The adsorption conditions were identical to those described in Section 2.2.2. The results showed that the biomaterial had a total chlorophyll content of 3.41 mg/g sorbent. The ratio of chlorophyll *a* to chlorophyll *b* was 2.7. In general, the chlorophyll content did not significantly affect (*p* > 0.05) the adsorption capacity, and the average value for the chlorophyll-free sorbent remained statistically similar to the pristine material (11.07 vs. 11.12 mg/g). These results pointed out that ENR adsorption did not occur through the formation of complexes, as occurs with other polycyclic planar structures, which are trapped by chlorophylls at their planar surfaces [35].

#### 2.2.8. Equilibrium Studies

Empirical adsorption isotherms are frequently used to understand the distribution of pollutants between liquid and solid phases at equilibrium. In this research, the experimental data were fitted using the linearized isotherm models of Langmuir, Freundlich, and Dubinin–Radushkevich (Figure S2). Table 3 shows the governing equations as well as the results of the isotherm parameters at temperatures of 288, 298, and 308 K. In general, the Langmuir isotherm model best fits the data, resulting in higher coefficient determination values (R^2^ > 0.9998), suggesting that the surface adsorption occurred in a monolayer [42]. It was also found that temperature significantly increased the adsorption capacity from 116.28 to 138.89 mg/g, increasing the temperature from 288 K to 308 K, respectively (Table 3). Moreover, the Langmuir constant (b) decreased from 0.011 to 0.005 L/mg as the temperature increased (from 288 K to 308 K), confirming that temperature significantly influenced the adsorbent–adsorbate interaction. Furthermore, the Dubinin–Radushkevich isotherm model was used to determine the type of sorption (physical or chemical). The sorption energy (E, kJ/mol) was calculated from the Dubinin–Radushkevich parameter β as follows:

(4) $$E=\frac{1}{\sqrt{2\beta }}$$

It is well known that physical, chemical (ion exchange/chemical sorption), and chemsorption activity occurs on active sites at E values of < 8, between 8 and 16, and between 20 and 40 kJ/mol, respectively. In this work, as shown in Table 3, the sorption energy values were in the range of 0.174 to 0.278 kJ/mol, indicating that adsorption is dominated by physical mechanisms.

Additionally, Table 4 shows the comparisons between the ENR sorption capacity of the tested material and that of the various sorbents assessed in the recent literature. As can be seen, the sorption capacity of the *P. koidzumii* sorbent was significantly higher than that of the majority of the sorbents, except for the lanthanide-integrated garlic peels. However, the combination of complex preparation processes, high energy/time consumption, and the environmental hazards of certain proposed methodologies to prepare the sorbents inevitably complicate its large-scale application.

#### 2.2.9. Thermodynamics

All thermodynamic factors, such as the change in Gibbs free energy (ΔG°, KJ/mol), the change in enthalpy (ΔH°, KJ/mol), and the change in entropy (ΔS°, KJ/mol K), were obtained with the following equations.

(5) $$\Delta G{}^{\circ}=-RT\ln k_{c}$$

(6) $$\ln k_{c}=\frac{\Delta S{}^{\circ}}{R}-\frac{\Delta H{}^{\circ}}{R\;T}$$

In the equations, R is the ideal gas constant (8.314 J/mol K), T is the temperature (K), and $k_{c}$ is the equilibrium constant.

As presented in Table 5, an increase in temperature decreases the ΔG° parameter, suggesting that the adsorption process becomes more favorable at higher temperatures. The positive value of ΔH° (9.605 kJ/mol) also confirms the endothermic nature of the process. Therefore, in endothermic reactions, there is a beneficial effect of increasing the temperature. Moreover, it is well known that the heat involved in physisorption is in the range of the heat of condensation (2–20 kJ/mol) [43]. In this work, the value of ΔH° (9.605 kJ/mol) falls within this range, confirming that physical mechanisms are involved during ENR sorption. Finally, the positive value of ΔS° (7.408 J/mol K) is indicative of increased randomness at the solid/liquid interface [44].

#### 2.2.10. The Proposed Adsorption Mechanism

**Figure 10:**
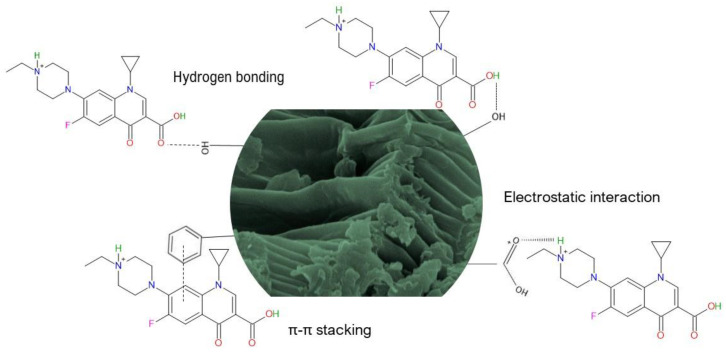
Mechanisms proposed for the adsorption of enrofloxacin onto *P. koidzumii* sorbent.

According to the results obtained with the adsorption kinetics, isotherms, thermodynamics, and the FTIR technique (before and after ENR adsorption), the interactions associated with the mechanism of ENR adsorption by using the *P. koidzumii* sorbent are schematically depicted in Figure 10. In general, three possible mechanisms could be involved during adsorption:

- Electrostatic interactions: the oxygen-containing functional groups were involved in ENR adsorption through electrostatic interactions. These functional groups on the sorbent surface could electrostatically bind to the amino group of the ENR molecule. Consequently, the electrostatic interactions may be the most important mechanism of the adsorption of ENR onto the *P. koidzumii* sorbent;
- Hydrogen bonding interactions: in the FTIR spectrum of the *P. koidzumii* sorbent after its interaction with ENR (Figure 1), the band at 3298 cm^−1^ shifted to 3291 cm^−1^, and the band at 1035 cm^−1^ shifted to 1023 cm^−1^. These frequency shifts may be attributed to the interaction between ENR and both the hydroxyl and ether groups, respectively. Consequently, hydrogen bonding can also play a dominant role in ENR adsorption;
- π-π orbital interactions: the significant reduction in intensity and shift to larger wavenumbers of the band at 1613 cm^−1^ confirmed the participation of aromatics present on the surface of the adsorbent on ENR adsorption. Thus, π-π orbital interactions are also involved in the ENR adsorption mechanism.

These interactions (electrostatic, hydrogen bonding, and π-π stacking) are mainly under the control of physical forces, and this fact was well corroborated by the ΔH° value (9.605 kJ/mol), which is in the range of the heat involved in physisorption.

#### 2.2.11. Reusability of Adsorbent

**Figure 11:**
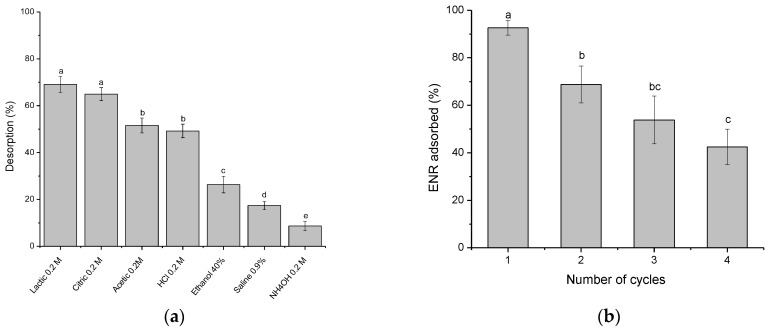
(**a**) Enrofloxacin desorption using different eluents, and (**b**) the reusability study of the *P. koidzumii* sorbent (adsorbent dose 8 g/L; 100 mg ENR/L; desorption time 40 min; and temperature 298 K). Those means that do not share a common superscript differ significantly (Tukey *p* < 0.05).

In order to make the adsorption process a more attractive alternative, desorption studies are essential for selecting an appropriate desorbing agent that allows the reuse of adsorbent and the recovery of the adsorbate. Based on ENR desorption results (Figure 11a), lactic and citric acid eluents enabled the highest ENR recovery over a short period of time (nearly 70%). However, for environmental and economic reasons, 0.2 M citric acid was chosen to evaluate the reusability of the sorbent. Four sorption–desorption cycles were conducted, and the results are shown in Figure 11b. In general, the fluoroquinolone uptake significantly decreased (*p* < 0.05) after the second adsorption–desorption cycle because some adsorption sites were unavailable for ENR uptake. From Figure 11b, it is observed that, after four cycles of use, the ENR adsorption decreased by more than 50%; consequently, the adsorbent could be reused for up to three cycles without significantly affecting its adsorption efficiency.

## 3. Materials and Methods

### 3.1. Adsorbate

The fluoroquinolone ENR was purchased from Bayer (Baytril^®^ Bayer Co, Shawnee Mission, KS, USA, 100 mg/mL). Some adsorbate properties are summarized in Table 6. Solutions were weekly prepared with Milli-Q water (resistivity 18.25 MΩ·cm at 298 K and a TOC value ≤ 5 µg/L), and the antibiotic concentration was determined using a fluorescence LS-55 spectrophotometer (Perkin Elmer, Waltham, MA, USA). The fluorescence emission spectra were collected in the wavelength range of 350–550 nm at an excitation wavelength of 272 nm. A quartz cuvette, with 1-cm path length, was used, and the excitation and emission slits were set at 10 nm, respectively. The ENR concentration was calculated using a standard reference (ENR, CAS number 93106-60-6, Merck KGaA, Darmstadt, Germany), with a calibration curve. The detection limit for ENR via fluorescence measurement is approximately 1.5 μg/L.

### 3.2. Adsorbent

Freshly pruned residues of *P. koidzumii* were collected at the beginning of the winter season in the Botanic Garden of the Superior Studies Faculty at Cuautitlan (National Autonomous University of Mexico). The method for sorbent production has been previously described in detail by Ramales-Valderrama et al. [30]. Briefly, the leaves were manually separated, washed thoroughly in running water to remove all traces of dust and dirt, rinsed with distilled water, and dried. Drying was carried out in a solar-energy drying system (designed and manufactured by UNAM-FESC) for 24 h at 323 K, with a drying airflow rate of 2 g/s. Then, the dehydrated leaves were carefully milled (C-11-1, Glen Mills Inc., Clifton, NJ, USA) and sieved to generate one particle size fraction of 60 mesh (≤250 µm).

### 3.3. Characterization

Functional groups on the surface of the adsorbent were characterized using a Frontier SP8000 Fourier transform infrared spectrophotometer (Perkin Elmer, Waltham, MA, USA), accessorized with an attenuated total reflection (ATR) attachment (DuraSamplIR II, Smiths Detection, Warrington, UK). Samples—before and after ENR adsorption—were placed on the ATR diamond crystal, and the spectra were acquired by co-adding 32 scans in the 4000–400 cm^−1^ region at a 4 cm^−1^ resolution. Proximate composition was carried out according to the procedures of the Association of Official Analytical Chemists (AOAC) [45]. The following analyses were performed in triplicate: moisture content (drying at 105 °C for 24 h); crude protein (micro-Kjeldahl, N × 6.25); crude fat (defatting in a Soxhlet equipment with hexane); total ash (incineration at 550 °C); and crude fiber (acid and alkaline hydrolysis), following the AOAC official methods 925.10, 960.52, 920.39C, 923.03, and 962.09E, respectively. A JEOL JSM-6510LV microscope coupled to an energy-dispersive X-ray microanalysis system (EDS INCA Energy 250 X-max 50, Oxford Instruments) was utilized for SEM-EDS characterization. A nitrogen sorption system (Micrometrics ASAP-2020, Norcros, GA, USA) was used to determine the specific surface area and porosity of the adsorbent. Zeta potential determinations were made at room temperature using the ZetaSizer Pro (Malvern Instruments, Worcestershire, UK). Measurements were performed on samples (5 mg dispersed in 5 mL Milli-Q water), adjusted to different pH values by the addition of HCl (0.1 M) or NaOH (0.1 M). Briefly, coarse particles were allowed to settle for 5 min in conical test tubes; subsequently, 100 µL of the aqueous phase was taken and diluted with 2 mL Milli-Q water to reduce the scattering and viscosity effects. Diluted samples were then analyzed in a disposable capillary cell DTS1070 at room temperature with an equilibration period of 120 s. A combination glass electrode (Conductronic PC-45, Puebla, Mexico) was used for monitoring pH. The isoelectric point (iep) was obtained by plotting zeta potential as a function of solution pH. Moreover, the point of zero charge (pH_pzc_) was assessed by adding equal amounts of sorbent to a set of flasks containing Milli-Q water at different pH values. Samples were shaken at 200 rpm for 40 min, and after settling, the pH value of the supernatant was determined. The pH_pzc_ was obtained from the plot of ∆pH against pH.

### 3.4. Adsorption Studies

Adsorption experiments were carried out in a batch system at room temperature (except for the temperature evaluation) in 100-mL flasks containing 50 mL of ENR solution. Flasks were shaken on a rotary shaker at 200 rpm. Immediately after sorption, the suspensions were centrifuged (7000× *g* for 7 min) and the supernatant was filtered through a PTFE membrane syringe filter (pore size 0.22 μm). The residual concentration of ENR was determined spectrofluorometrically at λ_em_ = 430 nm. All experiments were performed in quintuplicate. The adsorption capacity (mg/g) was calculated using the following equation.

(7) $$q_{e}=\frac{\left(C_{0}-C_{e}\right)\;V}{m}$$

where $C_{0}$ and $C_{e}$ are the initial and equilibrium concentrations of ENR (mg/L), V is the solution volume (L), and m represents the mass of the sorbent (g).

#### 3.4.1. The Effect of Different Factors on the Sorption of Enrofloxacin

Further experiments were conducted to investigate the factors influencing ENR adsorption. First, the sorbent dose was evaluated to find the optimal dosage on the basis of reaching the maximum adsorption rate. Thus, the optimal adsorbent dosage was selected for all subsequent trials, and the effects of other factors, such as contact time, solution pH, solution ionic strength, adsorbate concentration, and temperature, were analyzed. All the experimental conditions are as described in Table 7.

#### 3.4.2. Recycling and Regeneration Experiments

In order to investigate the reusability, the adsorbent was first used under the optimal conditions, and after centrifugation, the ENR-loaded sorbent was collected and transferred into seven desorbing agents: acetic acid (0.2 M), ammonium hydroxide (0.2 M), citric acid (0.2 M), ethanol (40% *v/v*), hydrochloric acid (0.2 M), lactic acid (0.2 M), and saline (0.9% *w/v*). The desorption experiments were conducted at room temperature for a period of 40 min at 200 rpm; afterward, the adsorbent was separated from the desorbing agent, washed with Milli-Q water, and dried in a vacuum oven at 50 °C in preparation for the next experiment. This procedure was repeated for up to four adsorption–desorption cycles.

### 3.5. Experimental Design and Statistical Analysis

The experiment was conducted as a completely randomized design, with five replicates. Before analysis, the data were confirmed for normality and homogeneity of variance by the Shapiro–Wilke and Levene tests, respectively. Then, the data were analyzed by one-way analysis of variance (one-way ANOVA), and means were separated using the Tukey honest significant difference post-hoc test, with the Minitab 16.0.1 software (Penn State University, State College, PA, USA). A significance value of α = 0.05 was used to distinguish significant differences.

## 4. Conclusions

This study presented a simple and ecofriendly methodology to prepare a low-cost sorbent from *P. koidzumii* leaves for the efficient removal of ENR from water. The biosorption of ENR greatly depends on adsorbent dosage, contact time, solution pH, solution ionic strength, adsorbate concentration, and temperature. The pseudo-second-order kinetic model provided the best fit to the experimental data, indicating that surface adsorption and diffusion into the pores participated in the adsorption. Moreover, the Langmuir isotherm model found the best fit with the data, suggesting that surface adsorption occurred in a monolayer. Furthermore, by means of the Dubinin–Radushkevich isotherm model, it was determined that the adsorption was dominated by physical mechanisms. Finally, the thermodynamic study revealed that the sorption of ENR becomes more favorable at higher temperatures. Based on these results, the pruning residues of *P. koidzumii* can be effectively used as an alternative adsorbent for the removal of ENR from water. However, further studies using a continuous column for the removal of different fluoroquinolone antibiotics from industrial and municipal effluents need to be conducted. Research in this direction is in progress in our laboratories.

## Figures and Tables

**Table 1:** Proximate composition of the *P. koidzumii* sorbent.

Component	%
Moisture content	6.29 ± 0.01
Crude protein	16.00 ± 0.08
Crude fat	2.02 ± 0.09
Total ash	5.08 ± 0.07
Carbohydrate *	70.61 ± 0.08

Mean values of three replicates ± standard error. * Including crude fiber.

**Table 2:** Kinetic parameters for the adsorption of enrofloxacin onto *P. koidzumii* sorbent.

Kinetic Model		Parameter *q_e_* (mg/g)	Enrofloxacin 11.12
Pseudo-first-order		*q_e_* (mg/g)	1.27
		*k_1_* (1/min)	0.029
		R^2^	0.9733
Pseudo-second-order		*q_e_* (mg/g)	11.18
		*k_2_* (g/mg min)	0.085
		R^2^	0.9999
Intraparticle diffusion		*k*_p_ (mg/g min^1/2^)	0.592
	Zone 1	*C* (mg/g)	8.04
		R^2^	0.9131
		*k_p_* (mg/g min^1/2^)	0.086
	Zone 2	*C* (mg/g)	10.27
		R^2^	0.7765
	Equilibrium	*q_e_* (mg/g)	11.01

**Table 3:** Isotherm parameters for the adsorption of enrofloxacin onto *P. koidzumii* sorbent.

Isotherm	Equation	Parameter	Temperature (K)		
			288	298	308
Langmuir	$\frac{C_{e}}{q_{e}}=\frac{C_{e}}{q_{m}}+\frac{1}{bq_{m}}$	*q_m_* (mg/g)	116.28	120.48	138.89
		*b* (L/mg)	0.011	0.008	0.005
		R^2^	0.9998	0.9990	0.9889
Freundlich	$ln\;q_{e}=ln\;K_{f}+\frac{1}{n}\;ln\;C_{e}$	*K_f_* (L/g)	4.377	3.382	2.890
		*n*	2.107	1.946	1.856
		R^2^	0.9144	0.9304	0.9242
Dubinin–Radushkevich	$ln\;q_{e}=ln\;q_{m}-\beta \epsilon^{2}$	*q_m_* (mg/g)	52.29	52.99	50.91
		E (kJ/mol)	0.278	0.204	0.174
		R^2^	0.6027	0.6212	0.5661

**Table 4:** Comparison of various studies for the adsorption of enrofloxacin onto some low-cost adsorbents.

Adsorbent	Modification	pH	Isotherm Model	Maximum Adsorption Capacity (mg/g)	Reference
Garlic peel (GP)	None	NR	Langmuir	0.65	[25]
	HNO_3_			9.89	
Garlic peel (GP)	HNO_3_-GP	7	Langmuir	29.8	[26]
	Tb@GP			580	
	Eu@GP			421	
	Tb/Eu@GP			769	
*Calotropis gigantea* fiber	NaClO_2_/acetic acid	6	Langmuir	62.93	[27]
Wheat bran	Acid-basic treatments	6	Sips	91.5	[28]
*P. koidzumii* leaves	None	7	Langmuir	138.89	This research

NR. Not reported.

**Table 5:** Thermodynamic parameters for the adsorption of enrofloxacin onto *P. koidzumii* sorbent.

Temperature (K)	ΔG° **(KJ/mol)**	ΔH° **(KJ/mol)**	∆S° (J/mol K)
288	7.435	9.605	7.408
298	7.361		
308	7.287		

**Table 6:** Some physicochemical properties of the enrofloxacin molecule.

Molecular Formula	Chemical Structure	Molecular Weight (g/mol)	pK_a1_ (Carboxyl)	pK_a2_ (Piperazinyl)	Fluorescence (nm) λ_ex/_λ_em_
C_19_H_22_FN_3_O_3_	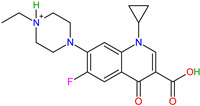	359.39	5.95	8.70	272/430

**Table 7:** The experimental set up for the adsorption of enrofloxacin onto *P. koidzumii* sorbent.

Item	Experimental Run	Enrofloxacin Concentration (mg/L)	Adsorbent Dose (g/L)	Contact Time (min)	Solution pH	Temperature (K)	Solution Ion Strength (M)
1	Sorbent dose	100	1–16	1440	7	298	-
2	Contact time	100	8	0–320	7	298	-
3	Solution pH	100	8	40	3–11	298	-
4	Ion strength	100	8	40	7	298	0.025 and 0.25
5	Adsorbate concentration	50–3200	8	40	7	298	-
6	Temperature	100	8	40	7	288–308	-

## Data Availability

The data presented in this study are available upon request from the corresponding author.

## Supplementary Materials

The following supporting information can be downloaded at https://www.mdpi.com/article/10.3390/antibiotics11111563/s1. Figure S1. EDS spectrum and EDS mapping of the *P. koidzumii* sorbent; Figure S2. Enrofloxacin adsorption isotherms at a temperature of 288, 298, and 308 K. (a) Langmuir, (b) Freundlich, and (c) Dubinin–Radushkevich models.
