# Supplementary material for: Outstanding Enrofloxacin Removal Using an Unmodified Low-Cost Sorbent Prepared from the Leaves of Pyracantha koidzumii
Source: Antibiotics (Basel). 2022 Nov 6;11(11):1563. doi: 10.3390/antibiotics11111563 (PMC9686792; doi:10.3390/antibiotics11111563)
Supplement: Supplementary file 1 [file antibiotics-11-01563-s001.zip › Figure S2.pdf]

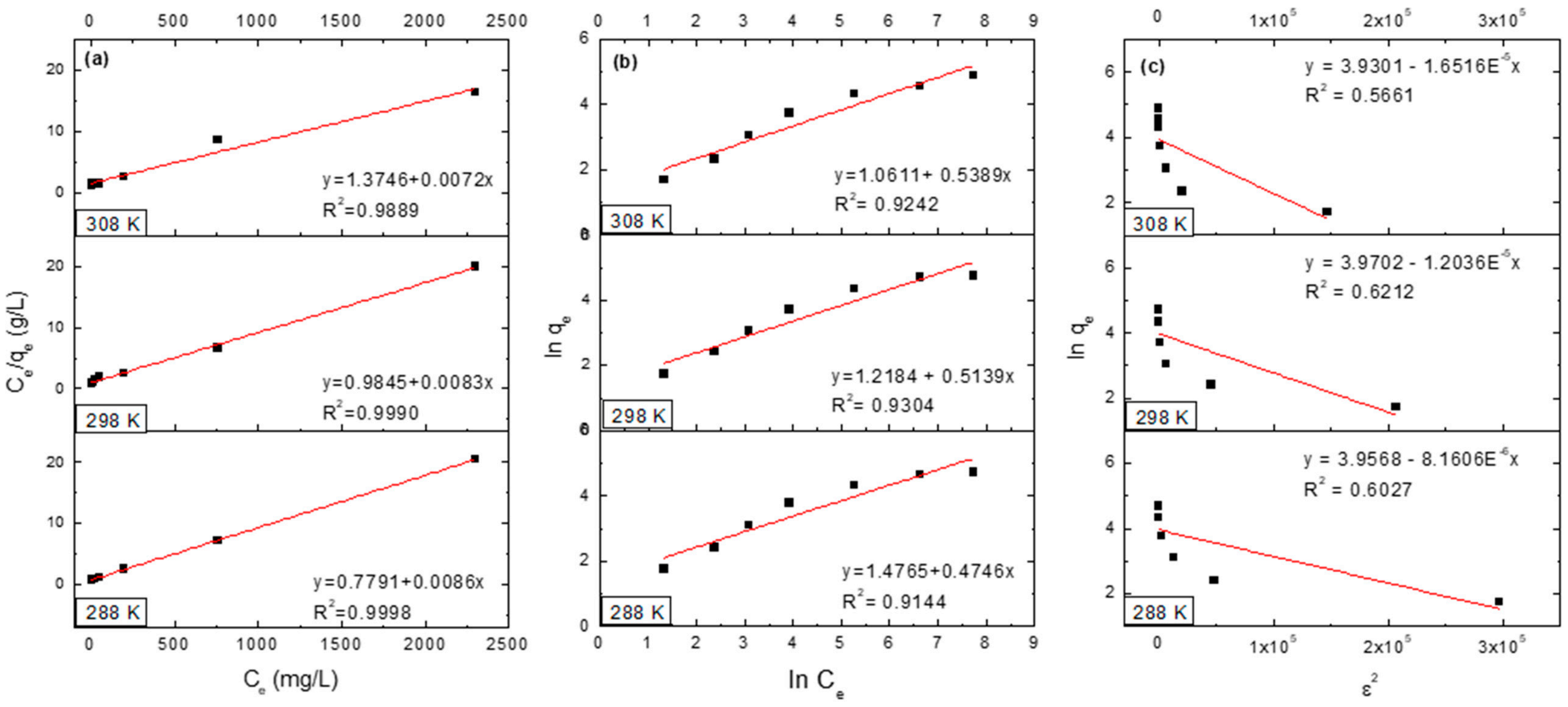

Figure S2. Enrofloxacin adsorption isotherms at a temperature of 288, 298, and 308 K. (**a**) Langmuir, (**b**) Freundlich, and (**c**) Dubinin–Radushkevich models.
