# Supplementary figures and images for: Outstanding Enrofloxacin Removal Using an Unmodified Low-Cost Sorbent Prepared from the Leaves of Pyracantha koidzumii
Source: Antibiotics (Basel). 2022 Nov 6;11(11):1563. doi: 10.3390/antibiotics11111563 (PMC9686792; doi:10.3390/antibiotics11111563)

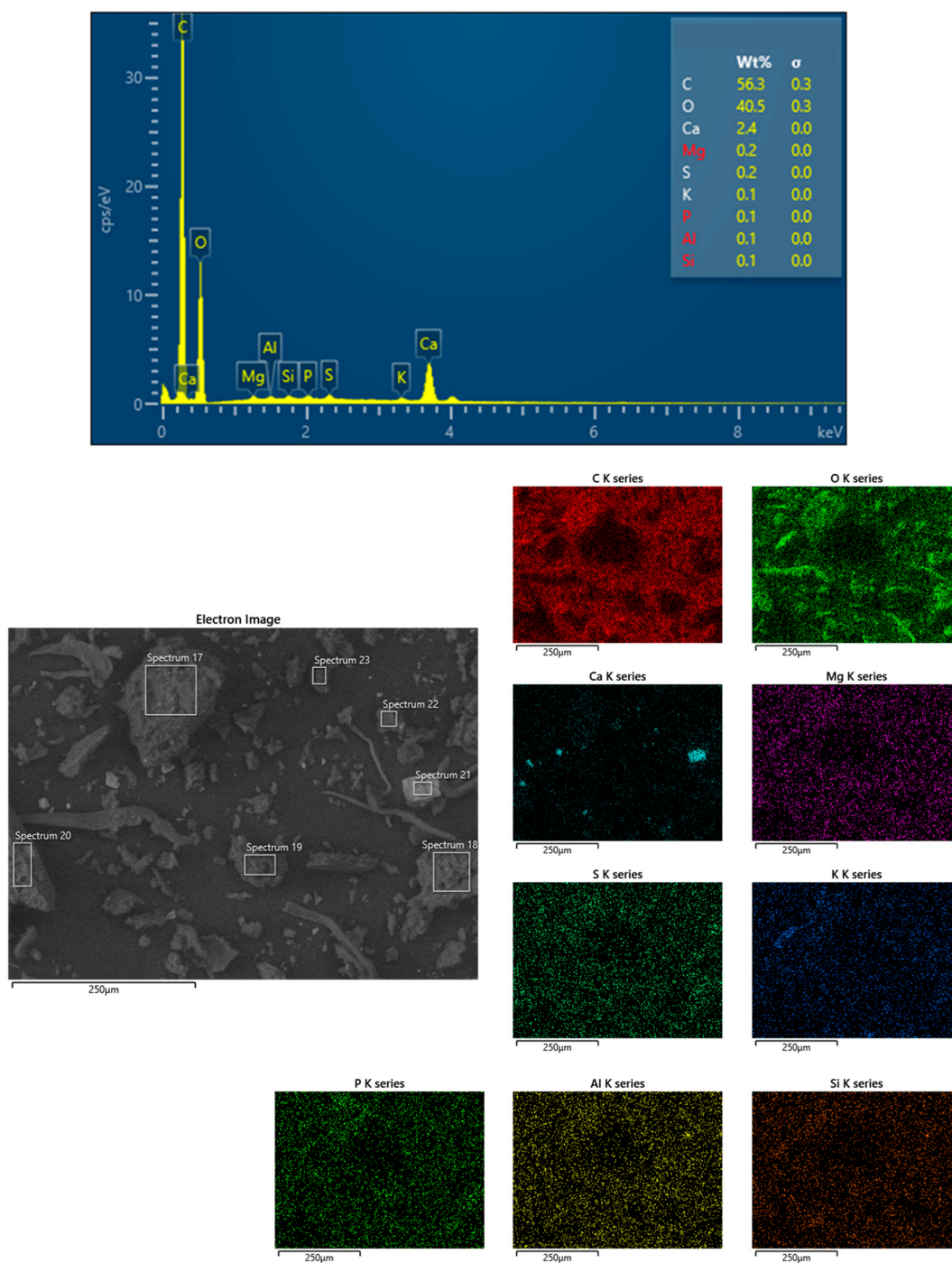

**Figure S1.** EDS spectrum and EDS mapping of the *P. koidzumii* sorbent.

Supplement: Supplementary file 1 [file antibiotics-11-01563-s001.zip › Figure S1.pdf]
